# The malaria testing and treatment market in Kinshasa, Democratic Republic of the Congo, 2013

**DOI:** 10.1186/s12936-016-1659-x

**Published:** 2017-02-28

**Authors:** ACTwatch Group, Angela Alum, Andrew Andrada, Julie Archer, Erick Auko, Katie Bates, Paul Bouanchaud, Meghan Bruce, Angela Camilleri, Emily Carter, Steven Chapman, Nikki Charman, Desmond Chavasse, Kevin Duff, Keith Esch, Anna Fulton, Kevin Duff, Keith Esch, Illah Evance, Anna Fulton, Hellen Gataaka, Tarryn Haslam, Emily Harris, Catharine A. Hurley, Beth Kangwana, Esther Kabui, Gloria Kigo, Aliza Lailari, Megan Littrell, Julius Ngigi, Kathryn A. O’Connell, Ricki Orford, Stephen Poyer, Justin Rahariniaina, Carolyne Ochieng, Linda Ongwenyi, Ricki Orford, Stephen Poyer, Justin Rahariniaina, Lanto Razafindralambo, Christina Riley, John Rodgers, Tanya Shewchuk, Julianna Smith, Tsione Solomon, Raymond Sudoi, Martine Esther Tassiba, Katherine Thanel, Andria Rusk, Julianna Smith, Rachel Thompson, Cynthia Whitman, Mitsuru Toda, Marie-Alix Valensi, Vamsi Vasireddy, Godéfroid Mpanya, Antoinette Tshefu, Joris Losimba Likwela

**Affiliations:** 1 Population Services International, 1120 19th St NW Suite 600, Washington, DC 20036 USA; 2 Association de Santé Familial, 4630 Avenue de la Science, Immeuble USTC, Bloc C, Gombe, Kinshasa, Democratic Republic of Congo; 3 Ecole de Santé Public de Kinshasa, Kinshasa, Democratic Republic of Congo; 4 National Malaria Control Programme, Kinshasa, Congo

**Keywords:** Democratic Republic of the Congo, Artemisinin-based combination therapy, ACT, Rapid diagnostic test, Market share, Availability antimalarial

## Abstract

**Background:**

The Democratic Republic of Congo (DRC) is one of the two most leading contributors to the global burden of disease due to malaria. This paper describes the malaria testing and treatment market in the nation’s capital province of Kinshasa, including availability of malaria testing and treatment and relative anti-malarial market share for the public and private sector.

**Methods:**

A malaria medicine outlet survey was conducted in Kinshasa province in 2013. Stratified multi-staged sampling was used to select areas for the survey. Within sampled areas, all outlets with the potential to sell or distribute anti-malarials in the public and private sector were screened for eligibility. Among outlets with anti-malarials or malaria rapid diagnostic tests (RDT) in stock, a full audit of all available products was conducted. Information collected included product information (e.g. active ingredients, brand name), amount reportedly distributed to patients in the past week, and retail price.

**Results:**

In total, 3364 outlets were screened for inclusion across Kinshasa and 1118 outlets were eligible for the study. Among all screened outlets in the private sector only about one in ten (12.1%) were stocking quality-assured Artemisinin-based Combination Therapy (ACT) medicines. Among all screened public sector facilities, 24.5% had both confirmatory testing and quality-assured ACT available, and 20.2% had sulfadoxine-pyrimethamine (SP) available for intermittent preventive therapy during pregnancy (IPTp). The private sector distributed the majority of anti-malarials in Kinshasa (96.7%), typically through drug stores (89.1% of the total anti-malarial market). Non-artemisinin therapies were the most commonly distributed anti-malarial (50.1% of the total market), followed by non quality-assured ACT medicines (38.5%). The median price of an adult quality-assured ACT was $6.59, and more expensive than non quality-assured ACT ($3.71) and SP ($0.44). Confirmatory testing was largely not available in the private sector (1.1%).

**Conclusions:**

While the vast majority of anti-malarial medicines distributed to patients in Kinshasa province are sold within the private sector, availability of malaria testing and appropriate treatment for malaria is alarmingly low. There is a critical need to improve access to confirmatory testing and quality-assured ACT in the private sector. Widespread availability and distribution of non quality-assured ACT and non-artemisinin therapies must be addressed to ensure effective malaria case management.

**Electronic supplementary material:**

The online version of this article (doi:10.1186/s12936-016-1659-x) contains supplementary material, which is available to authorized users.

## Background


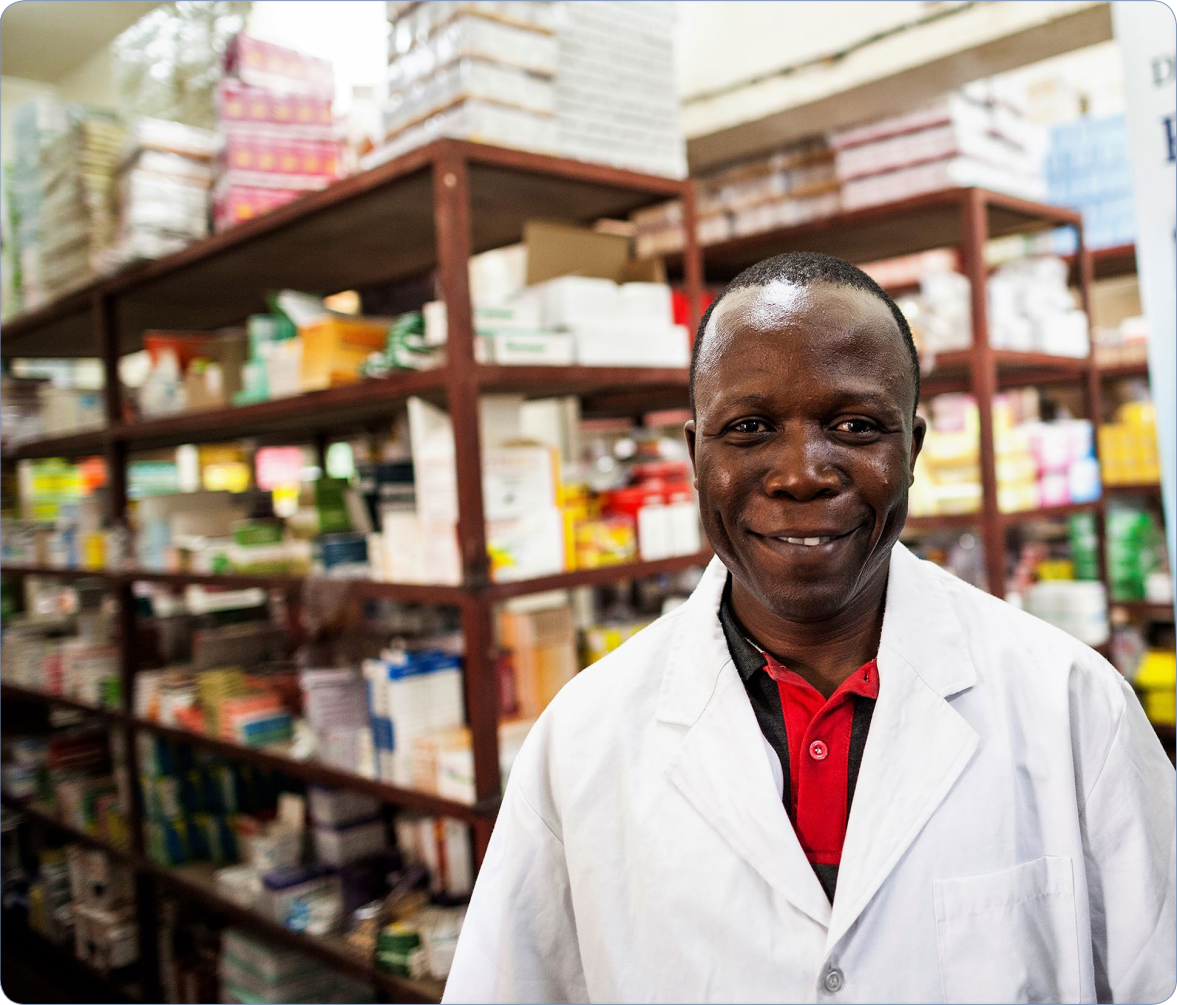
The Democratic Republic of Congo (DRC) is the second largest contributor to the global burden of disease due to malaria [1]. Malaria accounts for more than 40% of all outpatient visits and is the main cause of morbidity and mortality [1]. Of notable public health concern is the capital city of Kinshasa, which hosts up 15% of the DRC’s 79 million people [2]. Malaria prevalence is diverse given the geographical make-up of the city, with densely populated areas separated by large semi-rural areas, and with some of the areas completely rural in nature [3]. Recent evidence suggests malaria prevalence is around 11.9% in children 6–59 months, and as high as 31.7% in semi-rural areas of the city [3], illustrating not only the diversity of malaria prevalence and risk in Kinshasa, but that malaria is of grave public health concern in this populous area, a finding supported by earlier research [4, 5].

Ensuring that febrile patients in Kinshasa have access to confirmatory malaria testing and affordable artemisinin-based combination therapy (ACT)—the first-line treatment for uncomplicated malaria—will be central to ensuring patients are managed correctly in accordance with the national malaria control guidelines. The DRC malaria treatment guidelines state that all suspected cases should be confirmed by diagnostic testing prior to treatment. Positive cases of simple malaria should be treated with artesunate + amodiaquine (ASAQ) or artemether + lumefantrine (AL), ideally with a World Health Organization (WHO) pre-qualified product, referred to as a quality-assured ACT.

A 2009 survey conducted by the ACTwatch project [6] revealed that there are several barriers to appropriate treatment in the DRC—namely that availability of quality-assured ACT medicines in the private sector, where most people seek treatment, is low (Table 1) [7]. In 2009, only 14% of the anti-malarial stocking private sector had quality-assured ACT available. Furthermore, ACT medicines that were available in the private sector were namely non quality-assured. Oral artemisinin monotherapy was sold and distributed in the private sector, and contributed up to 10% of the total market share of all anti-malarials. Due to the threat that oral artemisinin monotherapy poses for the development of artemisinin resistance, this class of anti-malarial medicine was banned in the DRC in 2009 [1]. Finally, availability of malaria diagnosis was low in the private sector and restricted to private for-profit health facilities (21%). Availability was higher in the public sector, with around 80% of this sector stocking a diagnostic test.

**Table 1:** Key results from 2009 ACTwatch survey in Kinshasa

Dates of data collection	August–September 2009
**Sample**	**N**
Outlets screened	2368
Outlets interviewed	778
**Among all screened outlets, percentage that had**	**% (95% CI)**
Public sector availability of quality-assured ACT	48.5 (32.8, 64.6)
Private sector availability of quality-assured ACT	14.1 (10.6, 18.6)
Public sector availability of non quality-assured ACT	20.0 (11.4, 32.8)
Private sector availability of non quality-assured ACT	60.7 (55.2, 66.0)
Public sector availability of oral artemisinin monotherapy	26.0 (15.4, 40.3)
Private sector availability of oral artemisinin monotherapy	70.4 (65.4, 75.0)
**Anti-malarial market share**	**%**
Market share of oral artemisinin monotherapy	10.8

In 2013, a follow-up outlet survey was conducted in Kinshasa to understand the extent to which the anti-malarial landscape has changed over time. The paper provides a descriptive illustration of Kinshasa’s anti-malarial market as a means to understand current barriers regarding access to appropriate malaria case management and to guide future malaria interventions designed to improve access to affordable quality-assured ACT and confirmatory testing.

## Methods

The ACTwatch project is a multi-country research project implemented by Population Services International (PSI) and was launched in 2008 [6]. The ACTwatch project provides information on anti-malarial market evidence to inform case management strategies, and to monitor national and global policy and funding decisions [8, 9]. Outlet survey data are collected to understand the supply side of malaria medicine and diagnostic markets [10].

### Design and sample

A sub-national representative cross-sectional malaria medicine outlet survey was conducted in Kinshasa (11th September—11th October 2013). Methods for the surveys have been described in detail previously [6, 9]. Briefly, clusters with a population size of approximately 10,000 to 15,000 inhabitants, (“aires de santé”) were selected with probability proportional to size from a list of all clusters in Kinshasa province. Explicit stratification was used to sample from urban and rural areas of the province. All outlets with the potential to provide malaria medicine or testing were screened within each cluster to determine availability of malaria diagnostics and medicines. In the public sector, this included public health facilities and not-for-profit health facilities. In the private sector, this included private for-profit health facilities, pharmacies, drug stores, general retailers and itinerant drug vendors. Outlets were included in the survey if they had an anti-malarial in stock, or anti-malarials in stock in the past three months preceding the survey, or had malaria testing available.

The study was powered to detect a minimum of a 20% point change in availability of quality-assured ACT among anti-malarial stocking outlets with 80% power and 95% significance.

### Procedures

Outlets were identified using a census procedure within each of the selected clusters. All outlets were approached and administered a set of screening questions to determine eligibility. Outlets were eligible if they had an anti-malarial in stock on the day or survey or in the past three months. An interview with the staff member who was most likely to sell or prescribe medications was conducted. The interview was carried out in French or in local language (Lingala).

### Measures

A structured questionnaire included an audit of all available anti-malarials and malaria rapid diagnostic tests (RDTs). The audit captured product information including formulation, strength, brand name, active ingredients, manufacturer, and country of manufacture. Information was also collected on the retail price and the amount of the medicine sold in the last week, or number of RDTs performed in the past week. Availability, price and number of tests performed by microscopy was also collected.

### Data analysis

Double data entry was conducted using Microsoft Access (Microsoft Corporation, Redmond, Washington, USA) with built-in range and consistency checks. Data were analyzed across survey rounds using Stata (StataCorp College Station, TX). Stata survey settings were used to account for the stratified and clustered sampling strategy and to apply sampling weights. Sampling weights were calculated as the inverse of the probability of cluster selection.

Standard indicators were constructed according to definitions applied across the ACTwatch project and have been described elsewhere [20, 21]. For all key indicators, anti-malarials were categorized as ACT, non-artemisinin therapy or artemisinin monotherapy according to active ingredients. Artemisinin monotherapies were further classified as oral or non-oral to distinguish between the banned oral artemisinin monotherapies, and the non-oral artemisinin monotherapies, which may be used for treatment of severe malaria. ACT medicines were further classified as quality-assured or non quality-assured according to product information including active ingredients, strengths, manufacturer and country of manufacture. This product information was matched to lists of pre-qualified ACT medicines from the WHO and the Global Fund to identify quality-assured ACT that are manufactured according to global quality standards. Non quality-assured ACT medicines are defined as ACT medicines that do not have WHO pre-qualification or approval for procurement by the Global Fund.

Availability was determined according to the physical presence of anti-malarial medicines during the product audit. Anti-malarial availability was measured as the percent of outlets with each type of anti-malarial noted above in stock on the day of the survey. The volumes of the anti-malarials distributed were standardized using the adult equivalent treatment dose (AETD) to allow calculation of relative market share for types of anti-malarials, including tablet and all non-tablet dosage forms. The AETD is defined as the amount of active ingredient required to treat an adult weighing 60 kg according to WHO treatment guidelines [22]. Provider reports on the amount of the drug sold or distributed during the week preceding the survey were used to calculate volumes distributed. Relative market share was calculated as the amount of anti-malarial medicine distributed in the past week within each anti-malarial drug category (quality-assured and non quality-assured ACT, non-artemisinin therapy, oral artemisinin monotherapy, and non-oral artemisinin monotherapy), divided by the total anti-malarial distribution.

The AETD was also used for comparing the median private sector price for different anti-malarial medicines. Price was calculated for tablet formulation only given the difference in price across drug formulation. Provider reports for retail prices were converted to US dollars using official exchange rates. Median price and the inter-quartile range were calculated.

## Results

In total, 3364 outlets were screened for inclusion across Kinshasa and 1118 eligible outlets were interviewed and included in the analysis. Outlets stocking anti-malarials included public and private not-for-profit and for-profit health facilities, pharmacies, and drug stores. More than 2100 general retail outlets were screened, however anti-malarial medicines were not found in the general retail sector (Table 2). Of the outlets screened, 141 facilities had only diagnostic testing available and were not included in the analysis. Among outlets with anti-malarials in stock on the day of interview, 12,291 anti-malarial products were audited. In total, 278 RDT products were audited.

**Table 2:** Results of the outlet census

	N	Public sector		Private sector				
		Public facility	Private not-for-profit facility	Private for-profit facility	Pharmacy	Drug store	General retailer	Itinerant drug vendor
Outlets screened	3364	26	66	367	3	741	2149	12
Eligible outlets								
Anti-malarial in stock	962	21	41	174	3	722	1	0
Anti-malarial in stock in the past 3 months^a^	45	4	6	28	0	10	0	0
Malaria testing available^a^	142	4	16	122	0	0	0	0
Outlets interviewed								
Anti-malarial in stock in the past 3 months^a^	932	21	41	173	3	693	1	0
Malaria testing available^a^	45	1	6	28	0	10	0	0
Malaria testing available	141	4	16	121	0	0	0	0
Products audited								
Anti-malarials	12,291	109	169	815	85	11,111	2	0
Malaria RDTs	278	8	13	54	1	3	0	0

^a^No anti-malarials in stock on the day of the survey

All public and private health facilities and pharmacies and 98.6% of drug stores stocking anti-malarial medicines reportedly employed a provider who completed secondary school. In addition, all public health facilities and pharmacies, nearly all private not-for-profit (99.5%) and for-profit health facilities (99.7%), and 82.2% of drug stores reportedly employed a provider with a formal health qualification in medicine, pharmacy, nursing, midwifery or community health work (see Additional file 1: Table S1).

### Anti-malarial market composition

**Fig. 1:**
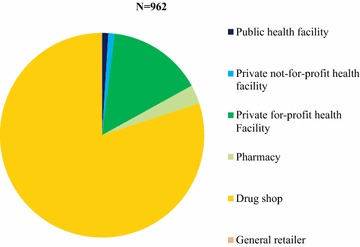
Anti-malarial market composition

Figure 1 shows the relative distribution of all outlets that had at least one anti-malarial in stock (n = 917). The anti-malarial market in Kinshasa was dominated by the private sector, and in particular by drug stores. Drug stores accounted for 80% of anti-malarial-stocking outlets in 2013, while private for-profit health facilities comprised 15% of the market. The public sector (public health facilities and private not-for profit facilities), accounted for less than 5% of the market composition.

### Availability of anti-malarials

**Fig. 2:**
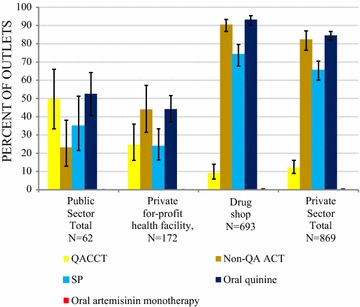
Anti-malarial availability among anti-malarial stocking outlets

Figure 2 shows availability of different classes of anti-malarials as the proportion of anti-malarial stocking outlets. Among the anti-malarial stocking public sector, 49.7% had quality-assured ACT in stock compared to 12.1% in the private sector. Within the anti-malarial stocking private sector, less than 10% of drug stores and only one in five private for-profit health facilities (24.7%) were found to stock quality-assured ACT. Availability of non quality-assured ACT medicines was much higher in the private sector. Over 80% of private sector outlets stocked non quality-assured ACT compared to 23.2% among public sector outlets. Non quality-assured ACT was commonly available in anti-malarial-stocking drug stores (90.5%), and private-for profit health facilities showed moderate availability (44.0%). Non-artemisinin therapies were more commonly available in the private sector (typically sulfadoxine-pyrimethamine [SP] 65.4%; oral quinine 84.6%) as compared with the public sector (SP, 34.9%; oral quinine 52.6%). Availability of oral artemisinin monotherapy was <1% in the private sector and 0% in the public sector.

### Anti-malarial market share

**Fig. 3:**
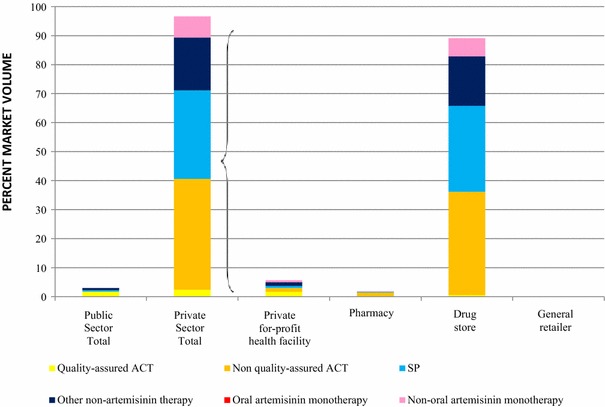
Anti-malarial market share

Figure 3 shows the relative anti-malarial market share for public and private sector and by type of anti-malarial medicine. Ninety-seven percent of all anti-malarials distributed to consumers in the week preceding the survey were distributed through the private sector. Most of the anti-malarials were distributed through drug stores (89.1% of the total anti-malarial market share).

Non-artemisinin therapies were the most commonly distributed anti-malarial (50.1% of the total market share). These included SP (31.1%) and quinine (19.0%). Non quality-assured ACT medicines accounted for 38.5% of all anti-malarials distributed. Quality-assured ACT medicines accounted for less than 5% of the total market share. Oral artemisinin monotherapy market share was <0.1%.

### Non quality-assured ACT in the private for-profit sector

**Fig. 4:**
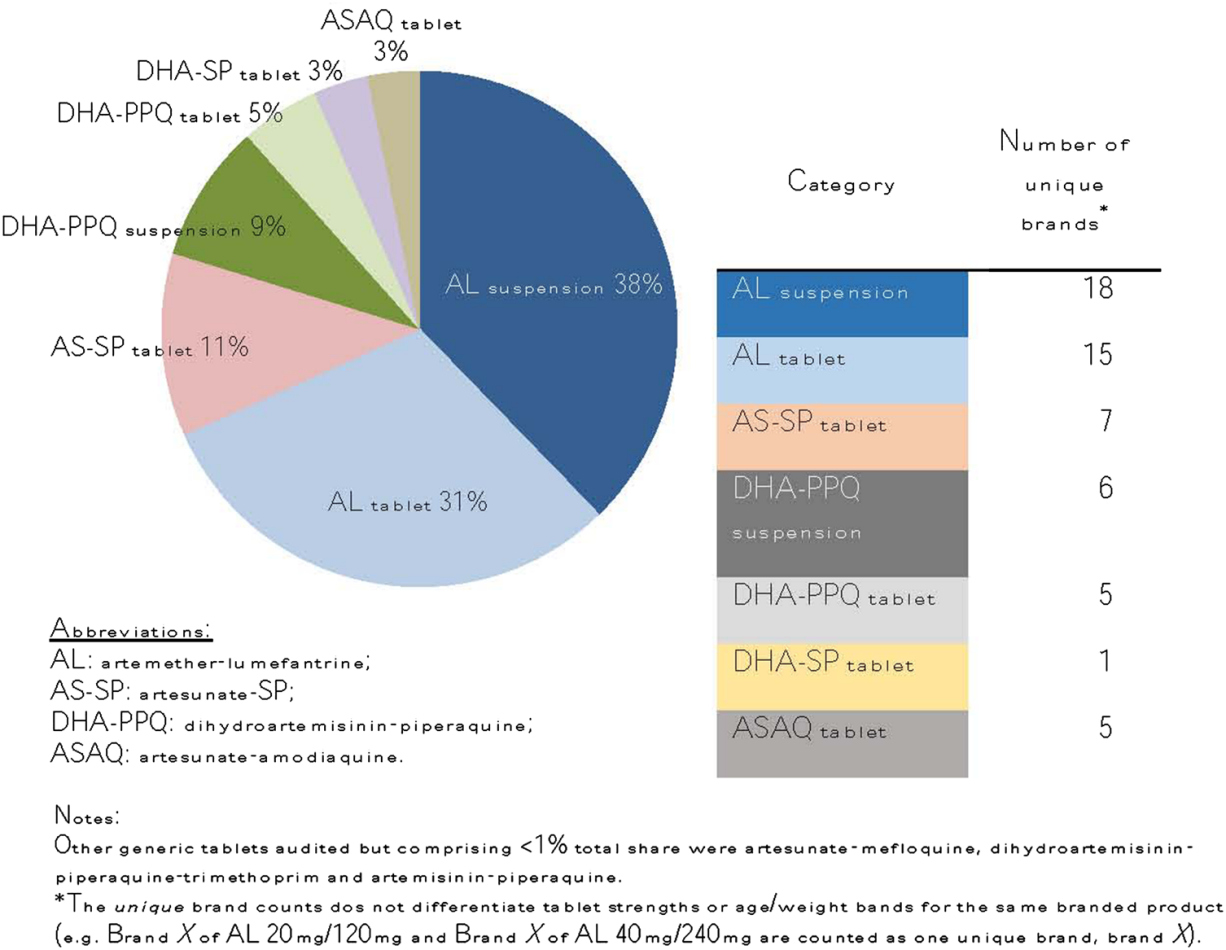
Distribution of non quality-assured ACT available in the private sector, 2013. *Notes* Types of non quality-assured ACT audited in private sector outlets by generic type and dosage form, Kinshasa 2013 (n = 5,982, unweighted). Other generic tablets audited but comprising <1% total share were artesunate-mefloquine, dihydroartemisininpiperaquine-trimethoprim and artemisinin-piperaquine. *AL* artemether-lumefantrine; *AS-SP* artesunate-SP; *DHA-PPQ* dihydroartemisinin-piperaquine; *ASAQ* artesunate-amodiaquine. *asterisk* The *unique* brand counts does not differentiate tablet strengths or age/weight bands for the same branded product (e.g. Brand *X* of AL 20mg/120mg and Brand *X* of AL 40mg/240mg are counted as one unique brand, brand *X*).

Figure 4 presents the frequency distribution by generic type and formulation of the 5982 non quality-assured ACT medicines audited in the private sector. Most non quality-assured ACT medicines available were artemether-lumefantrine (AL) suspensions (38%, 18 unique brands), AL tablets (31%, 15 unique brands) or suspensions (11%, 7 unique brands). Other types of non quality-assured ACT medicines included dihydroartemisinin-piperaquine tablets (5%, 5 unique brands) and suspensions (9%, 6 unique brands); as well as dihydroartemisinin-SP tablets (3%, 1 brand) and artesunate-amodiaquine tablets (3%, 5 unique brands).

### Price of anti-malarials

The median [inter-quartile range] price in drug shops for one adult equivalent treatment dose of quality-assured ACT was $6.59 [$2.75–$9.89], compared to $3.85 [$3.30–$4.94] for non quality-assured ACT. The median quality-assured ACT price in private for-profit facilities was $0.00, while non quality-assured ACT was on average sold at $3.71 [$2.75–$4.94]. SP was the cheapest tablet anti-malarial in the private for-profit sector ($0.44 [$0.33–$0.55] for one AETD).

### Readiness for malaria case management: availability of quality-assured ACT and malaria diagnosis, and availability of intermittent preventive therapy during pregnancy (IPTp)

Table 3 summarizes availability of key commodities for appropriate malaria case management and for provision of IPTp among all screened outlets (including outlets with and without anti-malarials on the day of the survey), as a means to show the extent to which different outlet types are able to provide nationally recommended case management commodities.

**Table 3:** Readiness for malaria case management in the public and private sector, among all screened outlets

	Percent (95% confidence interval [CI])			
	Total Public Sector	Private for-profit health facility	Drug store	Total Private Sector
	N = 92	N = 367	N = 741	N = 3272
Any malaria blood testing	86.8 (75.9, 93.2)	75.5 (64.9, 83.7)	0.3 (0.1, 1.5)	7.3 (6.1, 8.7)
Malaria microscopy	82.9 (72.0, 90.2)	72.9 (62.8, 81.1)	0.0	7.0 (5.8, 8.3)
RDT	21.8 (12.3, 35.8)	14.0 (10.3, 18.7)	0.3 (0.1, 1.4)	1.4 (1.0, 2.0)
Quality-assured ACT	28.7 (17.5, 43.2)	12.5 (8.2, 18.5)	8.6 (5.5 13.2)	3.3 (2.5, 4.4)
Readiness for malaria case management: *Malaria testing and quality*-*assured ACT available*	24.5 (15.0, 39.6)	10.7 (7.2, 15.6)	0.3 (0.1, 1.5)	1.1 (0.7, 1.7)
Readiness for IPTp: *SP available*	20.2 (11.7, 32.5)	12.0 (8.1, 17.5)	N/a	N/a

Public sector readiness for malaria case management was overall low with only one in four public sector outlets stocking both malaria testing and quality-assured ACT (24.5%). While availability of confirmatory testing was high (86.8%), availability of quality-assured ACT was much lower (28.7%). Availability of SP for ITPp was also low; only one in five public facilities had SP available on the day of the survey (20.2%).

In the private sector, only 1.1% of outlets had both confirmatory testing and quality-assured ACT available. Readiness was higher among private for-profit facilities (10.7%) as compared with drug stores (0.3%).

## Discussion

Data from a 2013 representative medicine outlet survey in Kinshasa confirm the importance of the private sector as a source of anti-malarial treatment, particularly drug stores. Results suggest urgent need to address malaria case management in the private sector, remove non quality-assured ACT medicines and inefective nonartemisinin therapies from the market, and address gaps in public sector readiness for appropriate case management.

### Urgent need to address malaria case management in the private sector

The distribution of anti-malarials in Kinshasa in 2013 was heavily dominated by the private sector, particularly drug stores which comprised almost 90% of the anti-malarial service delivery points. Drug stores in Kinshasa are not registered or regulated by a government authority, and are not authorized to test for malaria using RDTs. However, the vast majority of people receiving anti-malarial treatment in Kinshasa are receiving medicines from these unregistered and unregulated points of care where confirmatory testing is not permitted and quality-assured ACT treatment and malaria diagnostic testing is generally not available. With the vast majority of the antimalarial distribution moving through drug stores, these outlets most commonly stock and distribute non quality-assured ACT medicines and non-artemisinin therapies.

The finding that most of the anti-malarial medicine market share is comprised of drug stores in Kinshasa may provide an opportunity to increase access to affordable, quality-assured ACT medicines, as evidenced in other countries where these facilities are a common source of treatment [11–13]. The affordable medicines facility, malaria (AMFm), a large scale pilot to increase access to subsidized quality-assured ACT, demonstrated significant improvements in availability, affordability and relative market share for these medicines among the private sector and in drug stores in particular [14]. Since the AMFm pilot, follow-up ACTwatch surveys have shown a sustained improvement in the availability of quality-assured ACT medicines among drug stores in these countries [11–13]. The success of the private sector co-payment mechanism in these countries suggests that a similar strategy could be effective in improving the availability, affordability and market share for quality-assured ACT in Kinshasa.

Several countries have implemented successful interventions aimed at specifically strengthening drug stores for malaria case management. In Nigeria, Tanzania and Uganda, registration or accreditation programmes have been implemented in recent years to ensure that drug store providers are trained to assess and treat suspected malaria. For example, since 2006 the Tanzanian government has been in the process of accrediting drug dispensing outlets (ADDOs) to sell a limited range of prescriptions only medicines, including quality-assured ACT, after participation in a 35-day training programme. More recently, pilot initiatives have introduced RDTs into ADDOs with relative success, demonstrating patient willingness to pay for an RDT and an increased likelihood to purchase an ACT medicine [15]. Other research shows similar findings from a pilot test in Uganda where RDTs were introduced among drug stores that were licensed by the Ugandan Ministry of Health. The study concluded that by offering provider training and access to subsidized RDTs, it was possible to increase malaria testing rates significantly within communities [16]. These programmes demonstrate the feasibility of providing confirmatory testing and quality-assured ACT at these common points of care for suspected malaria, which is a common source of treatment in Kinshasa—as evidenced by other studies [17]. Approaches to engaging drug stores in Kinshasa with accreditation or other registration, training and supervision programmes could be promising for increasing coverage of confirmatory testing prior to treatment and improving access to quality-assured ACT.

### Widespread availability and distribution of inappropriate anti-malarial medicines

In 2009, the vast majority of anti-malarial-stocking outlets in Kinshasa were stocking oral artemisinin monotherapy. By 2013, oral AMT had disappeared from the market, demonstrating the success of the drug ban which had been implemented in 2009 [18].

Despite this achievement, multiple threats to effective malaria case management persist in the anti-malarial market in 2013. First is the widespread availability of non quality-assured ACT medicines. These are combination therapies that are not manufactured according to global standards set by the World Health Organization [19] and include up to 57 different brands with a spectrum of different formulations and active ingredients, though most commonly were either AL suspensions or tablets. While it was beyond the scope of this study to test the drug quality and efficacy of these products, the availability of multiple generics, formulations and brands which do not conform to global quality standards is of concern given the threats to effective parasite clearance and potential to contribute to artemisinin resistance.

The availability and distribution of non quality-assured ACT medicines is a growing problem documented in other countries by the ACTwatch project, including Kenya and Nigeria [20, 21]. However, levels of availability and distribution are much higher in Kinshasa and this is of particular concern given the significant malaria burden in the DRC. Non quality-assured products are commonly manufactured in the DRC and such locally-manufactured ACT medicines may also be prone to a number of risk factors for sub-standard quality, such as poor drug regulation and technical capacity in their development [22]. Given their lack of quality-assured status, these medicines have not been subjected to international good manufacturing practices and may have contents or dissolution times that are outside the acceptable limits due to poor quality control [23]. Other research that tested ACT formulations for their drug quality has shown that quality-assured ACT medicines have 0.1 times the odds of being poor quality compared with non quality-assured ACT (0.5% of quality-assured ACT tested were of poor quality compared to 5.4% of those non quality-assured ACT). Additional analysis revealed that products with quality-assured status remained significantly associated with anti-malarial medicine being of acceptable quality [24]. The results indicate that important improvements in quality can be achieved by ensuring that only products meeting WHO prequalification are registered and allowed on the market. The replacement of non quality-assured ACT with quality-assured ACT from the Kinshasa market will be important to ensure febrile children and adults have access to effective, first-line treatment.

The 2013 outlet survey also documented ongoing high levels of availability and distribution of non-artemisinin therapies, which are no longer recommended for uncomplicated malaria case management in the DRC. Non-artemisinin therapies accounted for half of all anti-malarials distributed in Kinshasa. These include oral quinine and SP. Although SP may be used for IPTp, availability and distribution through drug stores as opposed to health facilities, as well as product packaging and patient instructions on SP products promoting use for malaria case management for people of all ages, suggests that SP is being used as an inexpensive treatment for malaria [25]. The results also demonstrate that SP is far less expensive than quality-assured ACT in the private sector. These results point to the need to replace ineffective and non-recommended non-artemisinin therapies with quality-assured ACT medicines. The aforementioned AMFm pilot is one strategy which has shown promise in reducing stockage and distribution of non-artemisinin therapies, however challenges with availability and distribution of these products persist in the former AMFm countries given the relatively low cost of non-artemisinin monotherapies [26].

### Gaps in public and private sector readiness to manage malaria

The DRC was one of the first countries to adopt the second edition of the World Health Organization’s malaria treatment guidelines [27], for which a cornerstone of these guidelines was the recommendation to provide confirmatory diagnostic testing for all febrile patients, even in children under the age of five. Results from the 2013 outlet survey in Kinshasa demonstrate that nearly 90% of public facilities in Kinshasa have confirmatory testing available, primarily through malaria microscopy as opposed to RDTs. However, significant gaps persist in availability of quality-assured ACT, with fewer than one-third of public sector outlets stocking quality-assured ACT. Overall, only one in four public sector outlets have both confirmatory testing and quality-assured ACT available. Only one in five public outlets had SP available for IPTp during antenatal care visits.

Kinshasa’s readiness for appropriate malaria case management was extremely low in the private sector, and in particular among drug stores where most patients receive anti-malarial treatment. In drug stores, there was negligible availability of diagnostic tests and quality-assured ACT, pointing to the need to reinforce and promote the necessity of diagnostic testing among both providers and patients, while ensuring commodities are available and affordable within this sector as well as the public sector. Low availability of confirmatory testing in particular threatens appropriate management of suspected cases, spurring the potential for presumptive anti-malarial treatment. The low availability of confirmatory testing also merits a revisit of national policy to permit testing within these outlet types and provision of quality-assured treatment. As discussed previously, several initiatives in other countries have introduced RDTs into drug stores with relative success [15, 16], demonstrating the feasibility of providing confirmatory testing in the private sector.

### Implications

Given significant gaps in readiness for malaria case management documented in 2013, several measures were taken by the National Malaria Control Programme (PNLP), and with support from the Department For International Development (DFID), GFTAM, Association de Santé Familiale (ASF) and Population Services International (PSI), to transform the private sector anti-malarial market to improve quality malaria case management in Kinshasa [28]. This has included improving coordination between authorities and strengthening of the regulatory environment, establishing price reductions and increasing consumer demand for quality-assured ACT and fostering private sector case management. Over three and a half million quality-assured, subsidized ACT courses have been delivered over the course of the project and a further 1.7 million RDT. It is expected that future ACTwatch surveys will be able to document these achievements.

## Limitations

The data presented in this paper are representative for Kinshasa province only. Given the importance of the DRC with respect to global malaria burden, a key limitation is the lack of information on malaria markets in other areas of the DRC. Other limitations to the ACTwatch survey methodology have been described elsewhere in detail [10].

## Conclusions

While incredible progress has been made in recent years in improving access to malaria testing and quality-assured first-line treatment in many countries in sub-Saharan Africa [1], progress in the DRC has largely been absent. Given the significant burden of malaria in this country, there is urgent need to address gaps in both the public and private sector. Evidence from this study suggests that working with the private sector to remove non quality-assured ACT medicines and ineffective non-artemisinin therapies and to increase the availability and quality-assured ACT and confirmatory testing will be necessary to ultimately improve malaria case management in Kinshasa.

## Additional file

**Additional file 1: Table S1.** Provide demographics.

## Abbreviations

AETD: adult equivalent treatment dose
ACT: artemisinin-based combination therapy
AL: artemether-lumefantrine
ASF: Association de Santé Familiale
DFID: Department For International Development
DRC: Democratic Republic of Congo
IPTp: intermittent preventive therapy during pregnancy
PSI: Population Services International
RDT: rapid diagnostic test
