# Supplementary material for: The malaria testing and treatment market in Kinshasa, Democratic Republic of the Congo, 2013
Source: Malar J. 2017 Feb 28;16:94. doi: 10.1186/s12936-016-1659-x (PMC5330009; doi:10.1186/s12936-016-1659-x)
Supplement: Supplementary file 1 — Additional file 1: Table S1. Provide demographics. [file 12936_2016_1659_MOESM1_ESM.docx]

Supplementary Table S1: Provide demographics

| Percent of anti-malarial-stocking outlets with provider(s) that have completed secondary school and possess a health qualification | | | |
| --- | --- | --- | --- |
|  | N | Completed secondary school | Possess a health qualification* |
| Public Health Facility | 21 | 100.0 | 100.0 |
| Private Not-For-Profit Health Facility | 46 | 100.0 | 99.5  (97.2, 99.9) |
| Private For-Profit Health Facility | 171 | 100.0 | 99.7  (98.7, 100.0) |
| Pharmacy | 3 | 100.0 | 100.0 |
| Drug Store | 686 | 98.6  (97.3, 99.2) | 82.2  (75.6, 87.4) |

* Medical doctor, clinical officer, pharmacist, pharmacy technician, pharmacy assistant, nurse, nursing officer, medical assistant, nursing assistant, nursing aid, midwife, CHW
